# Brain-Derived Neurotrophic Factor Is Required for the Neuroprotective Effect of Mifepristone on Immature Purkinje Cells in Cerebellar Slice Culture

**DOI:** 10.3390/ijms20020285

**Published:** 2019-01-12

**Authors:** Jennifer Rakotomamonjy, Abdel Mouman Ghoumari

**Affiliations:** 1 Department of Physiology, Northwestern University Feinberg School of Medicine, 303 East Chicago Avenue, Chicago, IL 60611, USA; jennifer.rakotomamonjy@northwestern.edu; 2 INSERM UMR 1195 Inserm and Universities Paris-Sud and Paris-Saclay, 80 rue du Général Leclerc, 94276 Kremlin-Bicêtre, France

**Keywords:** Purkinje cells, mifepristone, BDNF, GABA_A_ receptors, MAP-kinase

## Abstract

Endogenous γ-aminobutyric acid (GABA)-dependent activity induces death of developing Purkinje neurons in mouse organotypic cerebellar cultures and the synthetic steroid mifepristone blocks this effect. Here, using brain-derived neurotrophic factor (BDNF) heterozygous mice, we show that BDNF plays no role in immature Purkinje cell death. However, interestingly, BDNF haploinsufficiency impairs neuronal survival induced by mifepristone and GABA_A_-receptors antagonist (bicuculline) treatments, indicating that the underlying neuroprotective mechanism requires the neurotrophin full expression.

## 1. Introduction

Purkinje cells die in cerebellar organotypic slice cultures when tissue is taken from animals between postnatal day (P)1 and P7, with a culminating point at P3 [1]. This period coincides with important developmental events such as neuronal migration and formation of synaptic connections [2,3]. In our previous study, we showed that Purkinje cell death following slice culture process was due, in part, to the γ-aminobutyric acid (GABA)-ergic network activity [4], known to be excitatory at this developmental stage [5,6]. When exposed to bicuculline, a GABA_A_ receptor antagonist, Purkinje cells survived [4]. This was the first demonstration that cerebellar slices from P3 rats or mice can release endogenous GABA, which induces strong GABAergic activity in the Purkinje cell network. We thus established that the neuroprotective effect of the synthetic steroid mifepristone [7] was due to its ability to depolarize Purkinje cell membrane potential to a value above chloride reversal potential, shunting GABAergic conductance [4,8]. 

Neurotrophins, such as brain-derived neurotrophic factor (BDNF), are essential for Purkinje cell development. BDNF is synthesized and released in an activity-dependent manner [9], and binds namely to its high-affinity receptor, tropomyosin receptor kinase B (TrkB) [10]. BDNF and TrkB are highly expressed in the postnatal cerebellum, including during the synaptogenesis stage [11,12]. They have been reported to promote dendritic growth [13,14], cerebellar development and foliation [15], Purkinje cell neuronal activity, and formation of functional neural circuits [16,17,18]. Besides these trophic effects, BDNF can also exacerbate excitotoxic insults to motor, cortical, and cerebellar neurons [19,20,21,22]. BDNF expressions markedly increase in many cell types following tissue injury, tissue insult, and aging [23,24]. Nevertheless, treating slice cultures of developmental mouse cerebellum with an antibody against BDNF or BDNF peptide did not affect the survival of Purkinje cells [25].

In developing neurons, BDNF expression is facilitated by GABA_A_ receptor activation [26]. In turn, the neurotrophin can modulate GABAergic activity [27,28]. BDNF has usually been identified as a potentiator of neurotransmitter release, GABA included [29], in diverse neurodegenerative contexts [30]. Given the mutual influence between GABA and BDNF, we sought to identify if the neurotrophin plays a role in immature Purkinje cell death in slice culture in synergy with, or independently of, GABA_A_ receptors activity and the neuroprotective effect of the steroid mifepristone. Our results show that, surprisingly, in our culture model, BDNF haploinsufficiency substantially decreases the neuroprotective effect of the steroid mifepristone on Purkinje neurons. This suggests that a full expression of BDNF is critical to prevent the neurotoxicity induced by the GABAergic network activity. 

## 2. Results

### 2.1. The Neurotrophin BDNF Is Not Involved in Immature Purkinje Cell Survival in Organotypic Cerebellar Slice Culture

**Figure 1:**
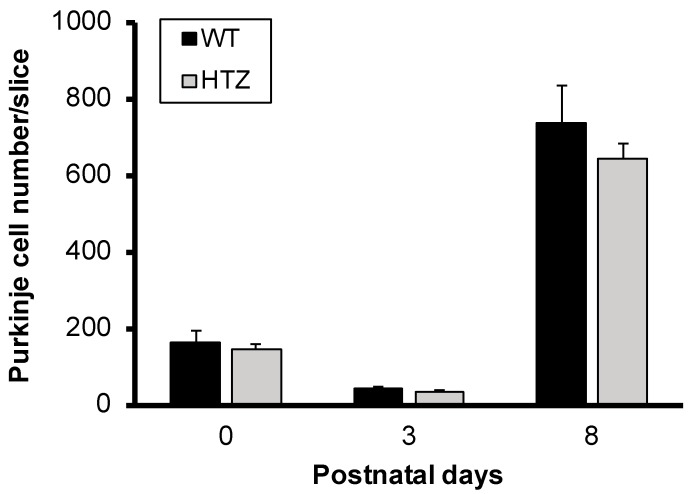
Brain-derived neurotrophic factor (BDNF) is not involved in immature Purkinje cell survival. Quantitative analysis of Purkinje cell survival in cerebellar slice culture from wild type (WT) or BDNF heterozygous (HTZ) mouse pups at different ages (postnatal-day 0: P0; -day 3: P3 and -day 8: P8).

To determine the involvement of BDNF in Purkinje cell survival in slice cultures, we first tested if the expression levels of BDNF could affect survival during postnatal development (P0–P8) in wild type (WT) and heterozygous (HTZ) BDNF mice. Cerebellar slices taken from P0, P3, and P8 WT and HTZ mouse pups were put in culture for 7 days without any treatment and then immunolabeled with the Purkinje cell marker, calbindin. At P0 we counted 162 ± 31 and 142 ± 13 Purkinje cells for WT and HTZ, respectively (Figure 1). Purkinje cell numbers decreased at P3 to reach 42 ± 6 and 35 ± 6 for WT and HTZ, respectively (Figure 1). Later at P8, Purkinje cells survival improved as we counted 735 ± 97 and 640 ± 43 Purkinje cells in WT and HTZ cerebellar slice culture, respectively (Figure 1). Our results show that BDNF expression levels do not affect Purkinje cell survival during the first postnatal week. 

### 2.2. BDNF Haploinsufficiency Impairs Neuroprotection by Bicuculline in Cerebellar Slice Culture

Previously, we have demonstrated that adding BDNF to slice cultures had no effect on Purkinje cell survival [25], presumably because of the difficulty for BDNF peptide to reach Purkinje cells in thick (350 µm) cerebellar slices. 

**Figure 2:**
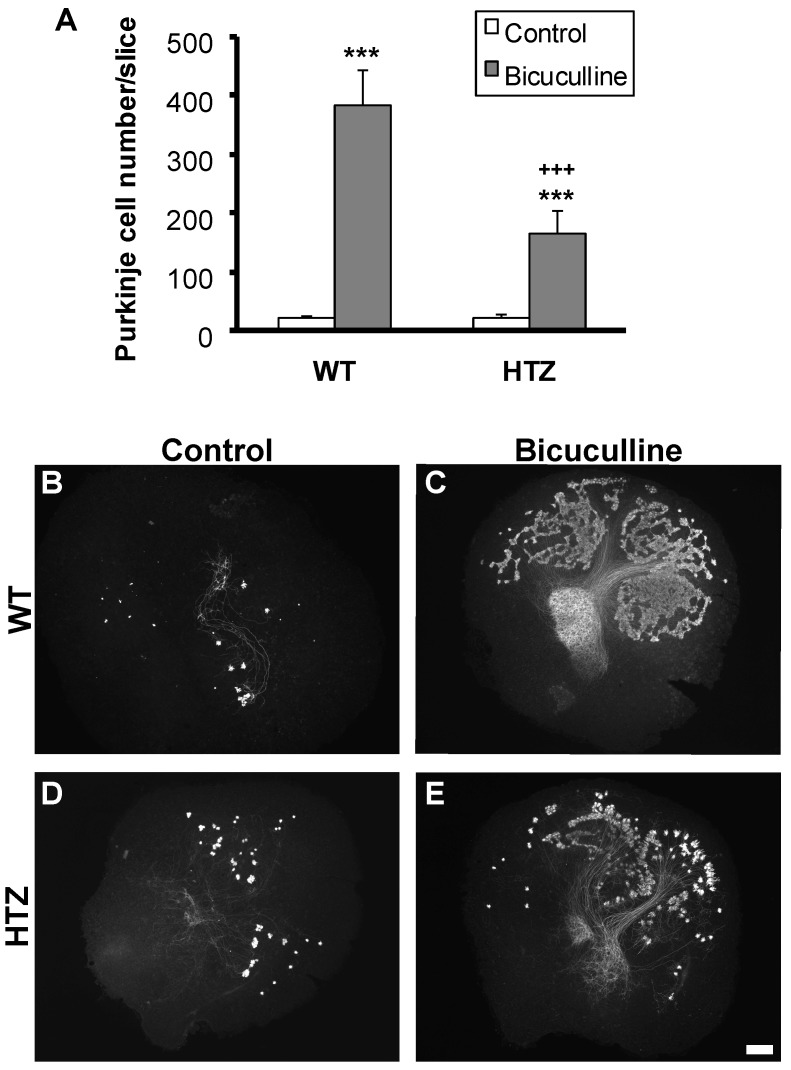
The neuroprotective effect of GABA_A_ receptor inhibition is half-lost in BDNF heterozygous mice. (**A**) Quantitative analysis of Purkinje cell survival in cerebellar slice cultures from wild type or BDNF heterozygous mice, treated with 100 µM bicuculline; (**B**–**E**) images of Purkinje cells labeled by Calbindin. (**B**,**D**) Control slices from wild type and BDNF heterozygous mice, respectively; (**C**,**E**) Bicuculline treated slices from wildtype and BDNF heterozygous mice, respectively. Scale bar, 200 µm. Data are expressed as mean of at least 3 independent experiments + SEM. *** *p* < 0.001 between control and treated slices, +++ *p* < 0.001 between WT and HTZ treated slices.

To investigate if BDNF is implicated in Purkinje cell death by GABAergic network neurotoxicity, cerebellar slices were taken from P3 wild type and BDNF heterozygous mice and put in organotypic cultures, treated or not with 100 µM bicuculline (GABA_A_-receptors antagonist). In WT mice, as previously shown [4], a large number of Purkinje cells survived with treatment (Figure 2A–C). Surprisingly, the neuroprotective effect of bicuculline showed a 57% decrease in HTZ mice. We counted 381 ± 62 Purkinje cells/slice in treated slices from WT mice, and 165 ± 39 Purkinje cells/slice in treated slices from HTZ mice (Figure 2A,D,E).

### 2.3. The Neuroptrophin BDNF Full Expression Is Necessary for Neuroprotection with Mifepristone

**Figure 3:**
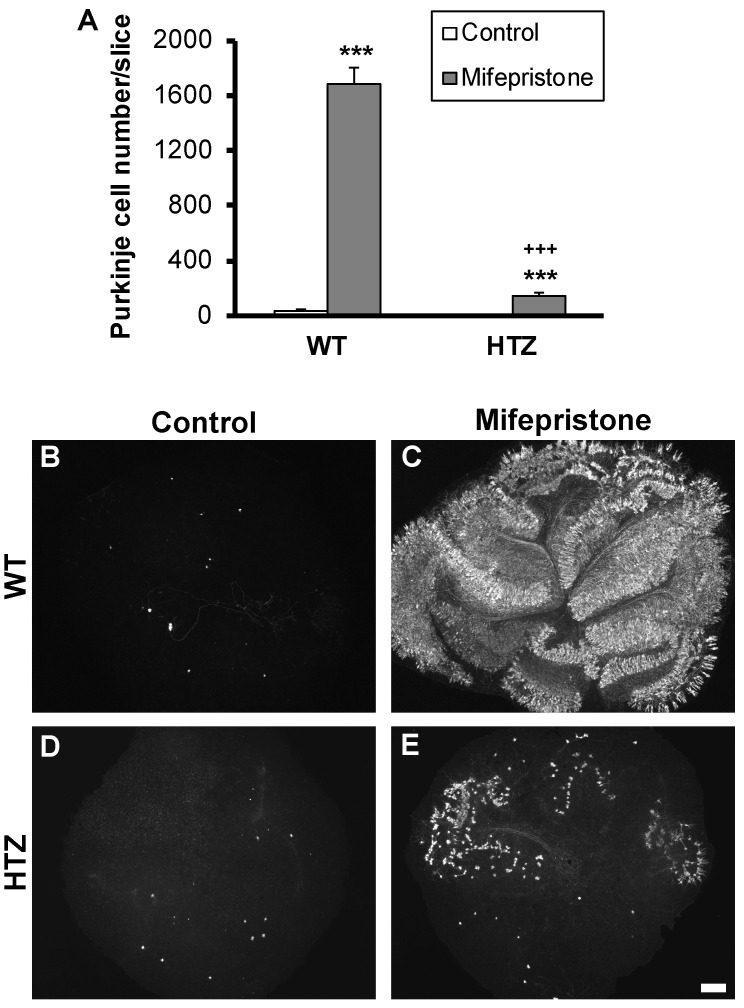
The neurotrophic factor BDNF is necessary for neuroprotection to occur with mifepristone. (**A**) Quantitative analysis of Purkinje cell survival in wild type or BDNF heterozygous mice treated or not with mifepristone (50 µM); (**B**,**D**) Control slices from wild type and BDNF heterozygous mice, respectively; (**C**,**E**) mifepristone treated slices from wild type and BDNF heterozygous mice, respectively. Scale bar, 200 µm. Data are expressed as mean of at least 3 independent experiments + SEM. *** *p* < 0.001 between control and treated slices. +++ *p* < 0.001 between WT and HTZ treated slices.

In light of these unexpected results obtained using bicuculline, we investigated if the synthetic steroid mifepristone, known to exert its neuroprotective effect via shunting of GABAergic conductance, would mimic bicuculline results. For this purpose, P3 cerebellar slices were taken from WT or BDNF HTZ mice and treated, or not, in organotypic cultures with 50 µM mifepristone. Next, we investigated if the decrease of endogenous BDNF could affect the survival of Purkinje cells under the steroid treatment. With the steroid, a large number of Purkinje cells survived (Figure 3A–C), as previously shown [7]. We counted 1689 ± 6 Purkinje cells in treated slices from WT mice, while slices from HTZ mice presented only 141 ± 33 Purkinje cells/slice (Figure 3D,E). 

### 2.4. Neuroprotective Effect of the p38 MAP-Kinase Inhibitor, SB203580, Is Not Affected by BDNF Haploinsufficiency

**Figure 4:**
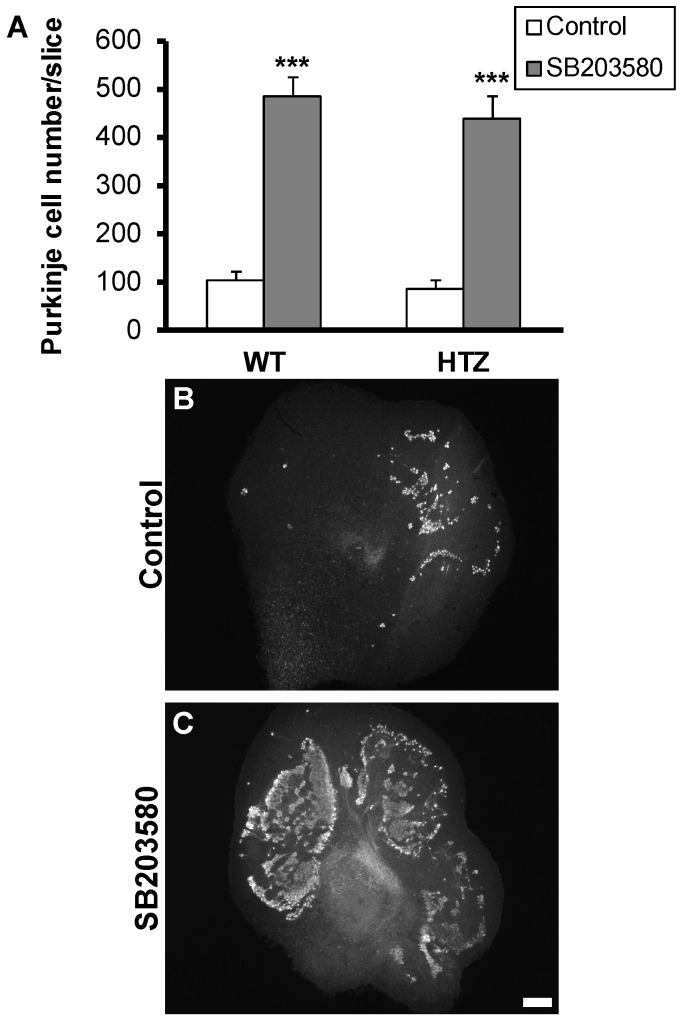
Neuroprotective effect of the p38 MAP-Kinase inhibitor, SB203580, is not affected by BDNF expression levels. (**A**) Quantitative analysis of Purkinje cell survival in wild type or BDNF heterozygous mice treated with SB203580 (20 µM); (**B**) Representative control slices from wild type or BDNF heterozygous mice; (**C**) Representative slice from wild type or BDNF heterozygous mice treated with SB203580 (20 µM). Scale bar, 200 µm. Data are expressed as mean of at least three independent experiments + SEM. *** *p* < 0.001 between control and treated slices.

We demonstrated in previous studies that Purkinje cell death involved p38 MAP-kinase signaling and that both neuroprotective treatments with bicuculline and mifepristone could prevent its activation [4]. Thus, we tested if p38 MAP-kinase signaling was BDNF-dependent. Cerebellar slice cultures were treated with the specific p38 MAP-kinase inhibitor SB203580 (20 µM) at P3. SB203580 induced high Purkinje cell survival as we counted about 106 ± 17 and 486 ± 40 Purkinje cells in untreated and SB203580 treated slices from WT mice, respectively (Figure 4). Furthermore, slices from HTZ mice presented 88 ± 18 and 438 ± 47 Purkinje cells/slice in untreated and SB203580 treated slices from HTZ mice, respectively (Figure 4). 

## 3. Discussion

During the postnatal period, the neurotrophin BDNF is highly expressed in the cerebellum, permitting its development and foliation as well as Purkinje cell dendritic growth and neuronal activity [11,12,13,14,15,16,17,18]. In the present study, we did not use Knock-out (KO) BDNF mice because of their early postnatal mortality. We used HTZ BDNF mice in cerebellar slice culture and found that the presence or absence of BDNF could not affect Purkinje cell survival during postnatal development (P0–P8), suggesting no role for BDNF in Purkinje cell survival in our model. These results confirm our previous studies, indicating that treating slices with an antibody against BDNF, or BDNF peptide, did not affect the survival of Purkinje cell [25]. One could suggest that BDNF is not implicated in Purkinje cell death. Surprisingly, the neurotrophin is absolutely needed for bicuculline- and mifepristone-induced Purkinje cell survival. This is consistent with other studies demonstrating the necessity of BDNF to support neuron survival [31]. For example, BDNF prevents the low potassium-induced-death of cultured cerebellar granule cells [32] and mediates the neuroprotective effect of estradiol on Purkinje cell following ethanol treatment [33]. In addition, the potential benefits of BDNF have been reported in several pathological conditions in other regions of the nervous system [34].

The relationship between BDNF expression and GABAergic transmission during central nervous system development are well documented [30]. GABA_A_ excitatory actions have been reported to increase BDNF expression [26]. Inversely, the neurotrophin can differentially modulate GABA_A_ transmission during development, in CA1 pyramidal neurons [28] and Purkinje cells [35]. It has also been reported that BDNF can increase the number of synapses from GABAergic neurons or the number of cell surface GABA_A_ receptors in cultures from rat visual cortex [36,37], or increase GABA release in a model of cortical neuron culture from postnatal day 2 rats [38]. Thus, synergistic actions between BDNF and GABA and their increasing releases could actually enhance Purkinje cell death in our model. 

Moreover, we previously showed that p38 MAP-kinase was activated after culture, peaking at 30 min, and downregulated by both mifepristone and GABA_A_ receptor inhibition [4]. Given this activation has been reported as necessary for in vivo BDNF production in rat hippocampus [39] or BDNF synthesis and release by microglia [40], it could represent a key factor in the neuroprotection process involving BDNF in our culture model. However, the neuroprotective effect of the pro-apoptotic MAP-kinase p38 inhibition does not need BDNF. It may be independent from the steroid mifepristone and GABA_A_ receptor inhibition effect underlying mechanisms, or the recruitment of MAP-kinase signaling would intervene downstream of BDNF actions. This is possible because previous studies demonstrated that BDNF could inhibit activation of p38 MAP-kinase and, thus, prevented cerebellar granule neurons death [41].

In summary, this is the first study showing a relevant intervention of neurotrophin BDNF in the mechanism of action of the steroid mifepristone. The presence of BDNF is not implicated in Purkinje cell death but it is necessary for the mifepristone neuroprotective effect. Hence, the final conclusion on a role of BDNF in the neuroprotective effect of mifepristone still requires further experiments to address how BDNF can impact on mifepristone-induced changes of Purkinje cell membrane polarity and on GABA-induced toxicity. 

## 4. Materials and Methods

### 4.1. Animals and Organotypic Slice Culture

All procedures were performed according to the European Communities Council Directive (2010/63/EU, adopted on 22 September 2010) for the care and use of laboratory animals. All mice were bred in our animal facility under a 12 h dark/light cycle with food and water *ad libitum.* BDNF +/− (HTZ) mice were purchased from Jackson laboratories (Bar Harbor, Maine, USA) and C57Bl/6 wild type (WT) mice from Janvier (Le Genest St Isle, France). Mouse pups at postnatal days 0 (P0), 3 (P3) and 8 (P8) were used for organotypic culture of cerebellar slices.

For all animals, after decapitation, brains were dissected out into cold Gey’s balanced salt solution with 5mg/mL glucose (GBSS-Glu, Sigma-aldrich, St. Quentin Fallavier, France), and meninges were removed. Parasagittal slices (350 µm thick) were cut on a MacIlwain tissue chopper and separated into cold GBSS-Glu. The slices were cultured on the membrane of a 30 mm Millipore culture insert (Millicell, Millipore, Bedford, MA, USA; pore size 0.4 µm) and maintained in culture 6-well plates containing 1 mL of medium at 35 °C in an atmosphere of humidified 5% CO_2_. The medium was composed of 50% basal medium with Earl’s salt, 25% Hank’s balanced salts solution, 25% horse serum (Invitrogen, Gaithersburg, MD, USA), L-glutamine (1mM), and 5 mg/mL glucose.

The principal chemical compounds used were the synthetic steroid RU486 (Mifepristone: 11β-(4-dimethylamino)phenyl-17β-hydroxy-17-(1-propynyl)estra-4,9-dien-3-one, Sigma), Bicuculline methiodide (GABA_A_-receptor antagonist, Sigma) and the MAP-kinase p38 inhibitor SB203580(4-[5-(4-Fluorophenyl)-2-[4-(methylsulfonyl)phenyl]-1*H*-imidazol-4-yl]pyridine, Bio-Techne Ltd., Lille, France). Cerebellar slices were exposed to these compounds only for the first two days of culture, and medium was replaced after two days without newly adding the respective steroids or drugs. Five days later, cultures were fixed for later immunostaining.

### 4.2. Immunofluorescence Analysis

For the detection of Purkinje cells, immunofluorescence for the calbindin D-28k was performed. The cultures were fixed in 4% paraformaldehyde in phosphate buffer (0.1 M), pH 7.4, for 1 h at room temperature. After washing in PBS, slices were taken off the Millicell and processed for immunohistochemistry. Slices were incubated for 1 h in a phosphate buffered saline blocking solution (0.12 M, pH 7.4) containing 0.9% NaCl, 0.25% Triton X-100, 0.1% gelatine, 0.1% sodium azide (PBSGTA) and lysine (0.1 M). Then, slices were incubated with rabbit polyclonal antibody against calbindin D-28k (1/10000, Swant, Bellinzona, Switzerland) in PBSGTA overnight at 4 °C. Slices were next incubated with secondary goat anti-rabbit CY3-labeled antibody (1/250 dilution, Jackson Immunoresearch Laboratories, Inc., West Grove, PA, USA) for 2 h at room temperature in PBSGTA. Slices were washed in PBS and then mounted in Fluoromount-G mounting medium (Clinisciences, France). Fluorescent images were acquired using a fluorescence microscope (Zeiss, Oberkochen, Germany), with the image analyzing system Axiovision 4 (Zeiss, Oberkochen, Germany).

To quantify the Purkinje cell survival in the cultures, each cerebellar slice culture was photographed and the total number of surviving Purkinje cells per slice was counted.

### 4.3. Statistical Analysis

Statistical parameters including the definitions and exact value of n, deviations, p values, and the types of the statistical tests are reported in the corresponding figure legends. Statistical analysis was carried out using Prism 6 (GraphPad Software). All statistical comparisons were conducted on data originating from at least three or more biologically independent experimental samples. Statistical comparisons between two groups were performed using an unpaired t test. Data are expressed as means ± SEM. Differences were considered significant with *p* < 0.05.
